# Outcomes of allogeneic SCT versus tisagenlecleucel in patients with R/R LBCL and poor prognostic factors

**DOI:** 10.1007/s12185-024-03888-9

**Published:** 2024-12-16

**Authors:** Kenta Hayashino, Toshiki Terao, Hisakazu Nishimori, Wataru Kitamura, Hiroki Kobayashi, Chihiro Kamoi, Keisuke Seike, Hideaki Fujiwara, Noboru Asada, Daisuke Ennishi, Keiko Fujii, Nobuharu Fujii, Ken-ichi Matsuoka, Yoshinobu Maeda

**Affiliations:** 1 Department of Hematology and Oncology, Okayama University Hospital, Okayama University, 2-5-1 Shikata-cho, Kita-ku, Okayama-shi, Okayama 700-8558 Japan; 2 Department of Hematology, Oncology and Respiratory Medicine, Dentistry and Pharmaceutical Sciences, Okayama University Graduate School of Medicine, Okayama, Japan; 3 Department of Hematology, Hiroshima City Hiroshima Citizens Hospital, Hiroshima, Japan; 4 Division of Blood Transfusion, Okayama University Hospital, Okayama, 2-5-1 Shikata, Okayama-shi, Japan; 5 Center for Comprehensive Genomic Medicine, Okayama University Hospital, 2-5-1 Shikata, Okayama-shi, Okayama Japan; 6 Division of Clinical Laboratory, Okayama University Hospital, Okayama, 2-5-1 Shikata, Okayama-shi, Japan

**Keywords:** Large B-cell lymphoma, Allogeneic hematopoietic stem cell transplantation, CAR-T cell therapy, Tisagenlecleucel, Poor prognostic factors

## Abstract

This study investigated the efficacy of tisagenlecleucel (tisa-cel) and allogeneic hematopoietic stem cell transplantation (allo-SCT) for patients with relapsed and/or refractory (r/r) large B-cell lymphoma (LBCL) with poor prognostic factors, defined as performance status (PS) ≥ 2, multiple extranodal lesions (EN), chemorefractory disease, or higher lactate dehydrogenase (LDH). Overall, the allo-SCT group demonstrated worse progression-free survival (PFS), higher non-relapse mortality, and a similar relapse/progression rate. Notably, the tisa-cel group showed better PFS than the allo-SCT group among patients with chemorefractory disease (3.2 vs. 2.0 months, *p* = 0.092) or higher LDH (4.0 vs. 2.0 months, *p* = 0.018), whereas PFS in the two cellular therapy groups was similar among those with PS ≥ 2 or multiple EN. Survival time after relapse post-cellular therapy in patients with poor prognostic factors was 1.6 with allo-SCT and 4.6 months with tisa-cel. These findings were confirmed in a propensity score matching cohort. In conclusion, tisa-cel resulted in better survival than allo-SCT in patients with poor prognostic factors. However, patients who relapsed post-cellular therapy had dismal outcomes regardless of therapy. Further strategies are warranted to improve outcomes in these patients.

**Supplementary Information:**

The online version contains supplementary material available at 10.1007/s12185-024-03888-9.

## Introduction

Diffuse large B-cell lymphoma (DLBCL) is a common subtype of B-cell non-Hodgkin’s lymphoma. Approximately 60–70% of patients with DLBCL who receive rituximab, cyclophosphamide, doxorubicin, vincristine, and prednisone achieve complete response (CR) [1, 2]. However, once they relapse, only 30–40% patients respond favorably to salvage chemotherapy and only 10% are cured after autologous stem cell transplantation (auto-SCT) [3]. For patients with relapsed or refractory (r/r) large B-cell lymphoma (LBCL), anti-CD19 chimeric antigen receptor (CAR) T-cell therapy, including tisagenlecleucel (tisa-cel), axicabtagene ciloleucel (axi-cel), and lisocabtagene maraleucel (liso-cel), have shown promising efficacy, with 30–40% patients achieving durable responses [4–6]. However, patients with specific clinical factors such as lower performance status (PS), multiple extra nodal (EN) lesions, chemorefractory disease before CAR-T cell infusion, and elevated lactate dehydrogenase (LDH) levels have been reported to demonstrate adverse survival outcomes after CAR-T cell therapy (median progression-free survival [PFS] ~ 3 months) [7–13]. Several previous studies have shown that these factors remain independent prognostic factors after multivariate analysis [9, 11, 13].

Allogeneic hematopoietic stem cell transplantation (allo-SCT) is an alternative curative treatment strategy for patients with r/r LBCL. Similar to patients receiving CAR-T cell therapy, the prognosis of patients with chemorefractory disease or non-CR status before allo-SCT is worse [14, 15]. For example, a previous study reported that the five-year overall survival (OS) was 23% in patients with non-CR status compared to 76% in those with CR status [15]. However, with the expansion of CAR-T cell therapy, the use of allo-SCT has decreased due to its higher transplant-related toxicity [16, 17]. Three retrospective studies were conducted to compare the safety and efficacy of these two curative cellular therapies. These studies reported that the CAR-T group had lower non-relapse mortality (NRM) and a higher relapse rate, and therefore, similar PFS [10, 18, 19], leading to the consideration of CAR-T cell therapy before allo-SCT in CAR-T-eligible regions [20]. Nevertheless, till date, no direct comparison has been made regarding whether CAR-T cell therapy is more effective or less toxic than allo-SCT in patients with poor prognostic factors.

To bridge this gap and contribute to further therapeutic improvement of CAR-T cell therapy and appropriate selection of cellular immunotherapy, we aimed to compare the outcomes of allo-SCT and tisa-cel, mainly focusing on poor prognostic factors for CAR-T cell therapy. This study provides valuable insights into the appropriate choice of cellular therapy in the CAR-T era.

## Materials and methods

### Study population

This retrospective study included all consecutive patients aged ≥ 18 years with r/r LBCL who underwent allo-SCT or received tisa-cel at Okayama University Hospital between January 2003 and May 2023. Patients who underwent both allo-SCT and CAR-T cell therapies were excluded. LBCL included the following entities: DLBCL not otherwise specified (DLBCL-NOS) or specific subtypes of DLBCL, including T cell/histiocyte-rich large B-cell lymphoma, intravascular large B-cell lymphoma, primary central nervous system lymphoma, transformed indolent B-cell lymphoma, high-grade B-cell lymphoma with MYC and BCL2 and/or BCL6 rearrangements, high-grade B-cell lymphoma not otherwise specified, or immunodeficiency-associated DLBCL. Patients with Burkitt lymphoma were not included in this analysis. Diagnoses were based on the 4th World Health Organization classification [21]. The clinical stage was defined based on the Ann Arbor system, and the International Prognostic Index (IPI) was determined at the initial diagnosis [22]. Conditioning regimens were divided into myeloablative conditioning (MAC) and reduced intensity conditioning (RIC) according to the previous report [23].

This study was approved by the Institutional Review Board of Okayama University Hospital (#2310-025) and conducted in accordance with the tenets of Declaration of Helsinki. This study information was disclosed on the website to provide an opt-out option. No participant provided a denial on study documents made available to them in an opt-out manner on the Web.

### Study definitions and endpoints

PFS was defined as the time from transplantation or CAR-T cell infusion to relapse, disease progression, death, or last follow-up. OS was defined as the time from transplantation or CAR-T cell infusion to the last date of follow-up or death. NRM was defined as the time until death without relapse or disease progression. The treatment response was based on the International Working Group response criteria [24]. Patients with the following characteristics were defined as those with “poor prognostic factors for CAR-T cell therapy” before commencement of cellular therapy: Eastern Cooperative Oncology Group-PS ≥ 2 (PS ≥ 2), EN ≥ 2, chemorefractory disease, or LDH ≥ upper limit of normal (ULN) [7–13]. Chemosensitivity was defined as partial response (PR) or CR before transplantation or infusion, whereas chemorefractory disease was defined as stable disease (SD) or progressive disease (PD). Disease status before transplantation or CAR-T cell infusion was classified into the following three types: CR, any CR; relapse, non-CR after achieving CR; and primary refractory, failure to achieve CR after commencing first treatment. LDH levels were measured immediately before conditioning or lymphodepleting chemotherapy. The overall response was defined as CR and PR with the best response. Primary endpoint was PFS. Secondary endpoints were OS, cumulative incidence of relapse/progression, and NRM.

### Statistical analysis

Patient characteristics were compared between the allo-SCT and tisa-cel groups. Categorical variables were analyzed using Fisher’s exact test. Continuous variables were assessed using Mann–Whitney U test. PFS and OS were estimated using Kaplan–Meier method, and groups were compared using the log-rank test. Time to relapse/progression and cumulative incidence of NRM were compared and calculated using Gray’s test. Major outcomes (PFS, OS, relapse/progression, and NRM) of patients with poor prognostic factors were compared. The outcomes were evaluated using univariate and multivariate Cox proportional hazards analyses. Moreover, to identify factors affecting relapse/progression, multivariate analysis was performed using the Fine-Gray proportional hazards regression based on factors with *p* < 0.05 in the univariate analysis as independent variables. Hazard ratios (HRs) with 95% confidence intervals (CIs) were calculated. NRM and relapse/progression were competing risks. Statistical significance was set at *p* < 0.05. To adjust the patient background between the tisa-cel group and the allo-SCT group, propensity score matching (PSM) was calculated in two patterns. First, we used age divided by 10-years and the number of prior regimens. Second, we used LDH levels and chemosensitivity. All statistical analyses were performed using EZR software (Ver. 1.61), which is a graphical user interface for R version 4.2 (The R Foundation for Statistical Computing, Vienna, Austria) [25].

## Results

### Patient characteristics

Patient characteristics (allo-SCT, n = 24; tisa-cel, n = 43) are summarized in Table 1. The allo-SCT group was significantly younger than the tisa-cel group (median age, 52.5 vs. 58.0, *p* = 0.035). The median number of prior regimens was 4 (range, 1–11) and the allo-SCT group had received significantly more prior regimens (median, 5 vs. 4; *p* = 0.016) than the tisa-cel group. There were no significant differences between the allo-SCT and tisa-cel groups in terms of sex, histology, stage, IPI, PS, history of autologous SCT, history of bone marrow or central nervous system involvement, the number of EN lesions, LDH level, disease status, and chemosensitivity. In the allo-SCT group, 10 patients (41.7%) underwent transplantation between 2003 and 2010, 14 (58.3%) between 2011 and 2020, and none after 2021. Twelve patients received RIC regimen. In contrast, in the tisa-cel group, no patients were treated between 2003 and 2010, 9 (20.9%) between 2011 and 2020, and 34 (79.1%) after 2021. The median follow-up time for survivors in the allo-SCT group was significantly longer than that in the tisa-cel group (80.2 vs. 12.5 months,* p* = 0.005).

**Table 1:** Patients characteristics

	Allo-SCT (n = 24)	Tisa-cel (n = 43)	*P* value
Median age, y (range)	52.5 (18–72)	58.0 (43–72)	0.035
Gender, n (%)			0.8
Male	12 (50)	24 (55.8)	
Female	12 (50)	19 (44.2)	
Disease histology, n (%)			0.38
DLBCL-NOS	11 (50)	28 (65.1)	
Transformed	7 (29.2)	10 (23.3)	
High grade B-cell lymphoma	1 (4.2)	0	
Immunodeficiency associated	2 (8.3)	3 (7)	
Other	3 (12.5)	2 (4.7)	
Stage, at diagnosis, n (%)			0.59
StageI/II	1 (4.2)	5 (11.6)	
StageIII/IV	22 (91.7)	37 (86)	
N/A	1 (4.2)	1 (2.3)	
IPI at diagnosis, n (%)			0.18
Low/Low-Int	13 (54.2)	12 (27.9)	
High-Int/High	10 (41.7)	21 (48.8)	
N/A	1 (4.2)	10 (23.5)	
PS at infusion			0.18
0,1	15 (62.5)	34 (79.1)	
2 ≥	8 (33.3)	9 (20.9)	
N/A	1 (4.2)	0	
Median No. of prior regimens (range)	5 (1–11)	4 (2–7)	0.016
Previous history of autologoous-SCT, n (%)	8 (33.3)	17 (39.5)	0.79
CNS invasion, n (%)	4 (16.7)	4 (9.3)	0.24
Bone marrow invasion, n (%)	13 (54.2)	14 (32.6)	0.051
EN lesion ≥ 2, n (%)	7 (29.2)	24 (55.8)	0.07
LDH ≥ ULN, n (%)	19 (79.2)	26 (60.5)	0.18
Disease status at infusion, n (%)			0.94
CR	4 (16.7)	7 (16.3)	
Relapse	12 (50)	19 (44.2)	
Primary refractory	8 (33.3)	17 (39.5)	
Response for last chemotherapy, n (%)			0.74
Chemosensitive	8 (33.3)	18 (41.9)	
Chemorefractory	16 (66.7)	24 (55.8)	
N/A	0	1 (2.3)	
Conditioning regimen, n (%)			N/A
MAC	12 (50)	N/A	
RIC	12 (50)	N/A	
Stem cell source, n (%)			N/A
BM	7 (29.2)	N/A	
PB	11 (45.8)	N/A	
CB	6 (25)	N/A	
Donor relation			N/A
Matched related	4 (16.7)	N/A	
Mismatched related	8 (33.3)	N/A	
Matched unrelated	4 (16.7)	N/A	
Mismatched unrelated	8 (33.3)	N/A	
Year of allo-HSCT or tisa-cell, n (%)			< 0.001
2003–2010	10 (41.7)	0	
2011–2020	14 (58.3)	9 (20.9)	
2021–2023	0	34 (79.1)	
Median follow up time in survivors, months (range)	80.2 (80–219.9)	12.5 (0.8–40.6)	0.005

*Allo-SCT* allogeneic hematopoietic stem cell transplantation; *auto-SCT* autologous hematopoietic stem cell transplantation; *BM* bone marrow; *CB* cord blood; *CNS* central nerve system; *CR* complete response; *DLBCL-NOS* diffuse large B-cell lymphoma not otherwise specified; *EN* extranodal; *IPI* International Prognostic Index; *LDH* lactate dehydrogenase; *N/A* not available; *MAC* myeloablative conditioning; *PB* peripheral blood; *PS* performance status; *RIC* reduced intensity conditioning; *tisa-cel* tisagenlecleucel; *ULN* upper limit of normal

### Survival outcomes and subgroup analysis with poor prognostic factors for CAR-T cell therapy

**Fig. 1:**
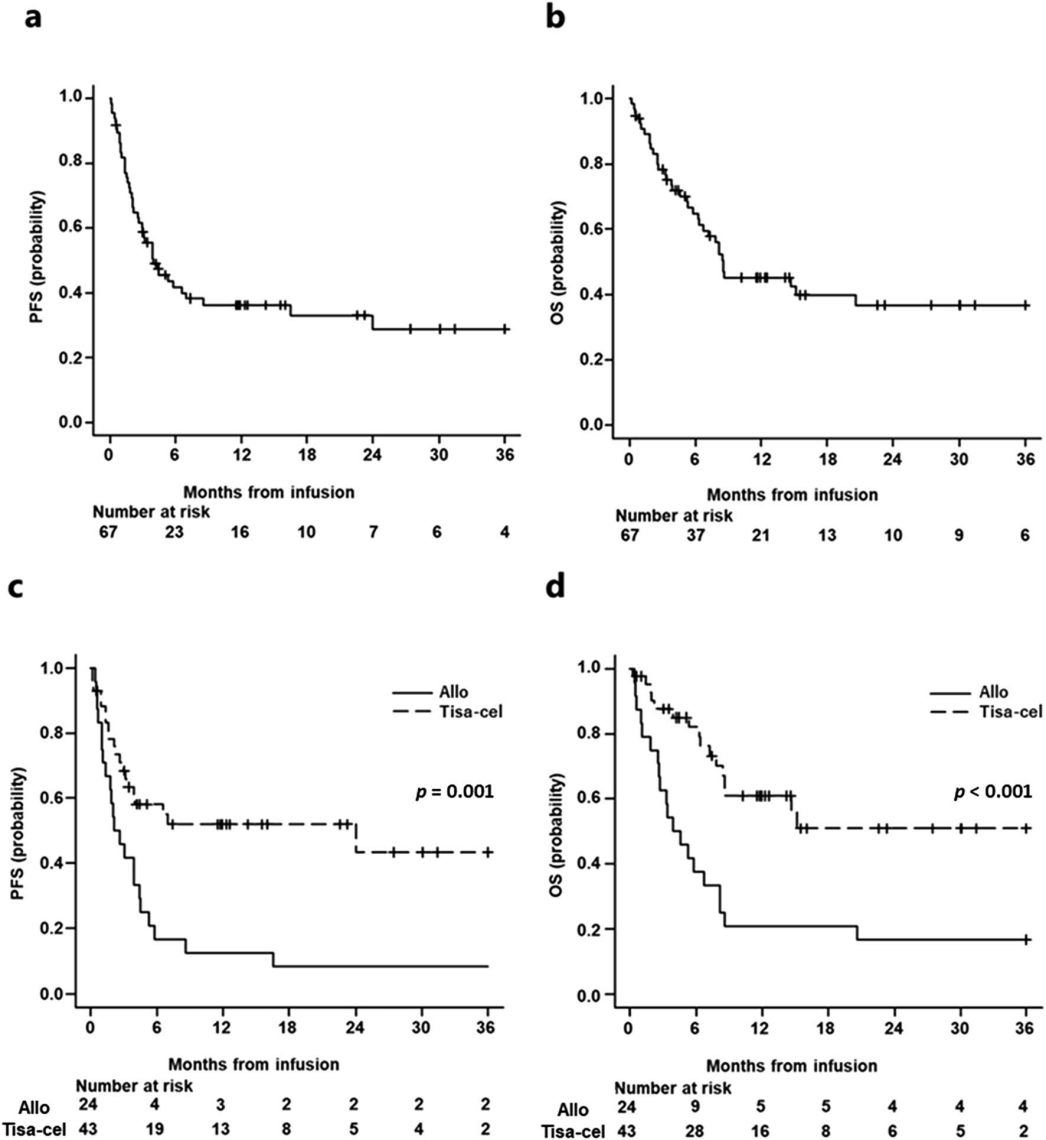
Survival outcomes. PFS (**a**) and OS (**b**) in all LBCL. Patients treated with tisa-cel had significantly better PFS (**c**) and OS (**d**)

The median PFS and OS of the entire r/r LBCL cohort (n = 67) were 4.0 and 8.5 months, respectively (95% CI: 2.6–8.5 months and 6.2–153.6 months) (Fig. 1a, b). The allo-SCT group demonstrated significantly worse median PFS and OS than the tisa-cel group (2.3 vs. 24.0 months and 4.2 months vs. not reached (NR), 95% CI: 1.1–4.4 months vs. 3.1-NR and 2.5–8.1 months vs. 8.4-NR, *p* = 0.001 and < 0.001, respectively) (Fig. 1c, d). Patients treated with tisa-cel showed better overall response than those treated with allo-SCT (74.4% vs. 58.3%, *p* = 0.27).

**Fig. 2:**
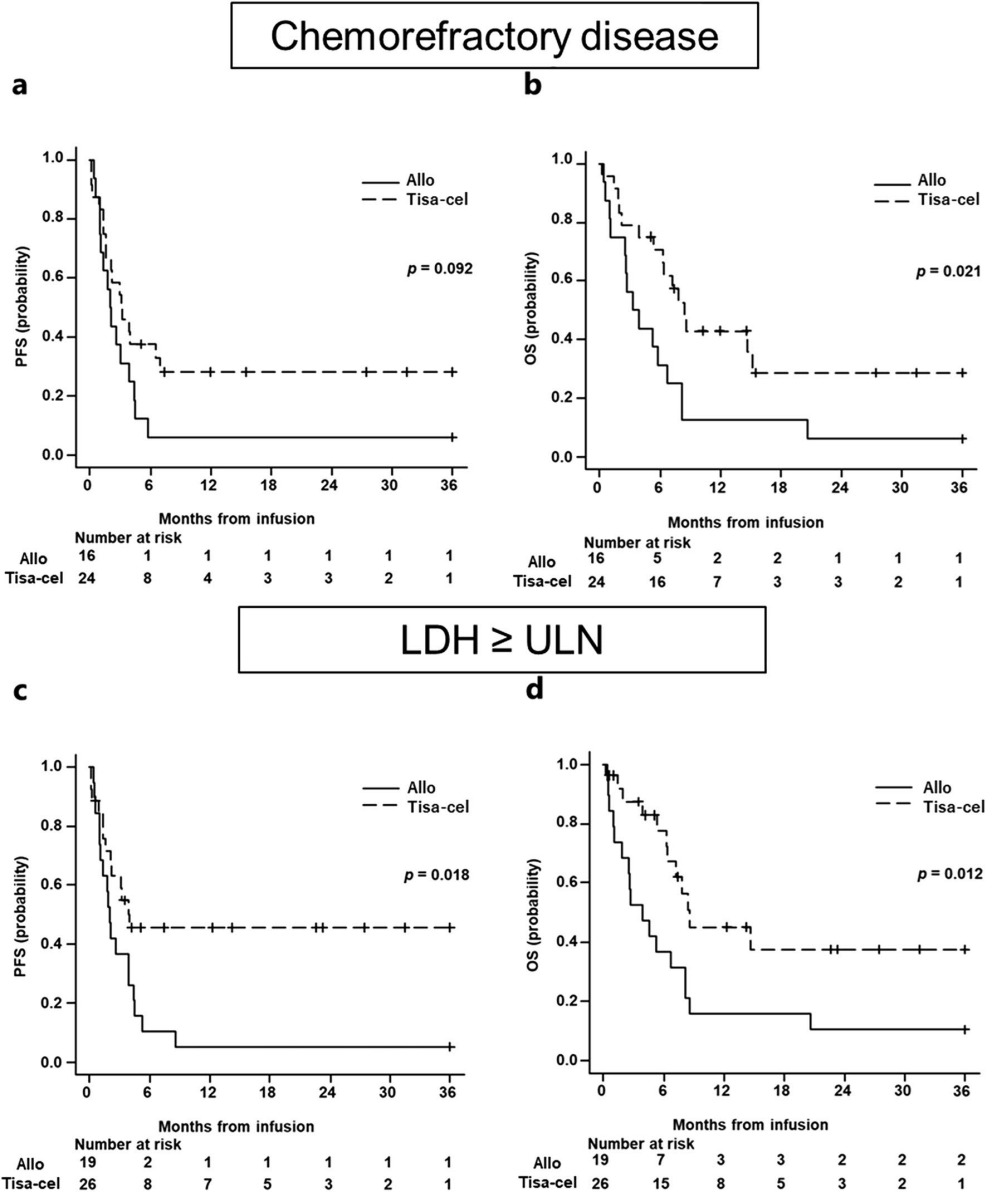
Survival outcomes with poor prognostic factors for CAR-T cell therapy. The patient treated with tisa-cel had better PFS (**a**) and OS (**b**) with chemorefractory disease, and PFS (**c**) and OS (**d**) with LDH ≥ ULN

Subgroup analysis was performed for patients demonstrating poor prognostic factors for CAR-T cell therapy in the context of the following parameters: PS ≥ 2 (all-SCT n = 8, tisa-cel n = 9), EN ≥ 2 (allo-SCT n = 7, tisa-cel n = 24), chemorefractory disease (allo-SCT n = 16, tisa-cel n = 24), and LDH ≥ ULN (allo-SCT n = 19, tisa-cel n = 26). Among the aforementioned four factors, patients with chemorefractory disease or LDH ≥ ULN showed better PFS and OS when receiving tisa-cel than allo-SCT (chemorefractory disease: median PFS in allo-SCT vs. tisa-cel group; 2.0 vs. 3.2 months [95% CI: 1.0–3.9 months vs. 1.5–6.9 months,* p* = 0.092] and median OS; 3.6 vs. 8.4 months [95% CI: 1.1–6.7 months vs. 5.3-NR, *p* = 0.021]) (LDH ≥ ULN: median PFS; 2.0 vs. 4.0 months [95% CI: 0.95–4.4 months vs. 1.5-NR, *p* = 0.018] and median OS; 3.9 vs. 8.5 months [95% CI: 1.1–8.1 months vs. 6.2-NR, *p* = 0.012]) (Fig. 2a–d). However, patients with PS ≥ 2 and EN ≥ 2 showed no significant differences in terms of PFS and OS between the allo-SCT and tisa-cel groups (PS ≥ 2: median PFS in the allo-SCT vs. tisa-cel group; 1.9 vs.1.6 months [95% CI: 0.66–3.9 vs.0.13-NR, *p* = 0.56] and median OS; 3.6 vs. 5.3 months [95% CI: 0.95–8.1 vs. 0.23-NR, *p* = 0.99]) (EN ≥ 2: median PFS; 2.0 vs. 3.2 months [95% CI: 0.95–4.5 months vs. 1.5–24.0 months, *p* = 0.40] and median OS; 2.7 vs.7.9 months [95% CI: 0.95–8.1 months vs. 3.8-NR, *p* = 0.14]). Moreover, survival outcome after relapse post-cellular therapy in patients with poor prognostic factors was 1.6 months (range 0.16–6.0 months) in the allo-SCT group and 4.6 months (1.7–6.3 months) in the tisa-cel group (Table 2).

**Table 2:** Survival outcomes with poor prognostic factors

	Number of patients	Median PFS (month, 95% CI)	*P* value	Median OS (month, 95% CI)	*P* value
All cases					
Allo-SCT	24	2.3 (1.1–4.4)		4.2 (2.5–8.1)	
Tisa-cel	43	24.0 (3.1-NR)	0.001	NR (8.4-NR)	< 0.001
PS ≥ 2					
Allo-SCT	8	1.9 (0.66–3.9)		3.6 (0.95–8.1)	
Tisa-cel	9	1.6 (0.13-NR)	0.56	5.3 (0.23-NR)	0.99
EN ≥ 2					
Allo-SCT	7	2.0 (0.95–4.5)		2.7 (0.95–8.1)	
Tisa-cel	24	3.2 (1.5–24.0)	0.40	7.9 (3.8-NR)	0.14
Chemorefractory disease					
Allo-SCT	16	2.0 (1.0–3.9)		3.6 (1.1–6.7)	
Tisa-cel	24	3.2 (1.5–6.9)	0.092	8.4 (5.3–NR)	0.021
LDH ≥ ULN					
Allo-SCT	19	2.0 (0.95–4.4)		3.9 (1.1–8.1)	
Tisa-cel	26	4.0 (1.5-NR)	0.018	8.5 (6.2-NR)	0.012

*Allo-SCT* allogeneic hematopoietic stem cell transplantation; *EN* extranodal; *LDH* lactate dehydrogenase; *NR* not reached; *OS* overall survival; *PFS* progression free survival; *PS* performance status; *tisa-cel* tisagenlecleucel; *ULN* upper limit of normal

To validate the prognostic impact of these two factors (chemorefractory disease and LDH ≥ ULN), we further performed univariate and multivariate analyses. In the univariate analysis of PFS, tisa-cel was associated with a favorable prognosis (HR 0.39, 95% CI: 0.21–0.73, *p* = 0.0028), while chemorefractory disease and LDH ≥ ULN were identified as adverse prognostic factors (HR 2.7, 95% CI: 1.3–5.4, *p* = 0.005 and HR 2.1, 95% CI: 1.0–4.0, *p* = 0.036, respectively). In multivariate analysis, tisa-cel remained significantly associated with improved prognosis (HR 0.43, 95% CI: 0.23–0.8, *p* = 0.0075), whereas chemorefractory disease was significantly associated with poorer prognosis (HR 2.4, 95% CI: 1.1–4.9, *p* = 0.02) (Table 3). In the univariate analysis of OS, tisa-cel demonstrated a favorable prognostic impact (HR 0.34, 95% CI: 0.17–0.65, *p* = 0.0013), while chemorefractory disease and LDH ≥ ULN were identified as adverse prognostic factors (HR 3.5, 95% CI: 1.5–8.0, *p* = 0.0032 and HR 2.7, 95% CI: 1.2–6.0, *p* = 0.012, respectively). Multivariate analysis revealed that tisa-cel was significantly associated with a superior prognosis (HR 0.37, 95% CI: 0.19–0.72, *p* = 0.0039), whereas chemorefractory disease was significantly associated with an unfavorable prognosis (HR 3.0, 95% CI: 1.2–7.2, *p* = 0.015) (Table 4).

**Table 3:** Univariate and multivariate analyses for PFS

	Univariate			Multivariate		
	HR	95% CI	*P* value	HR	95% CI	*P* value
Treatment: Tisa-cel	0.39	0.21–0.73	0.0028	0.43	0.23–0.80	0.0075
Chemosensitivity: Chemorefractory disease	2.7	1.3–5.4	0.005	2.4	1.1–4.9	0.02
LDH ≥ ULN	2.1	1.0–4.0	0.036	1.4	0.68–2.8	0.38

*CI* confidence interval; *HR* hazard ratio; *LDH* lactate dehydrogenase; *OS* overall survival; *PFS* progression-free survival; *tisa-cel* tisagenlecleucel; *ULN* upper limit of normal

**Table 4:** Univariate and multivariate analyses for OS

	Univariate			Multivariate		
	HR	95% CI	*P* value	HR	95% CI	*P* value
Treatment: Tisa-cel	0.34	0.17–0.65	0.0013	0.37	0.19–0.72	0.0039
Chemosensitivity: Chemorefractory disease	3.5	1.5–8.0	0.0032	3.0	1.2–7.2	0.015
LDH ≥ ULN	2.7	1.2–6.0	0.012	1.5	0.64–3.5	0.35

*CI* confidence interval; *HR* hazard ratio; *LDH* lactate dehydrogenase; *OS* overall survival; *PFS* progression-free survival; *tisa-cel* tisagenlecleucel; *ULN* upper limit of normal

In contrast, only 7 patients were without poor prognostic factors (allo-SCT n = 1, tisa-cel n = 6). The median follow-up time is 16.3 months (range, 3.1–80.2 months), and all of them are alive without relapse.

### Relapse/progression of disease and NRM

**Fig. 3:**
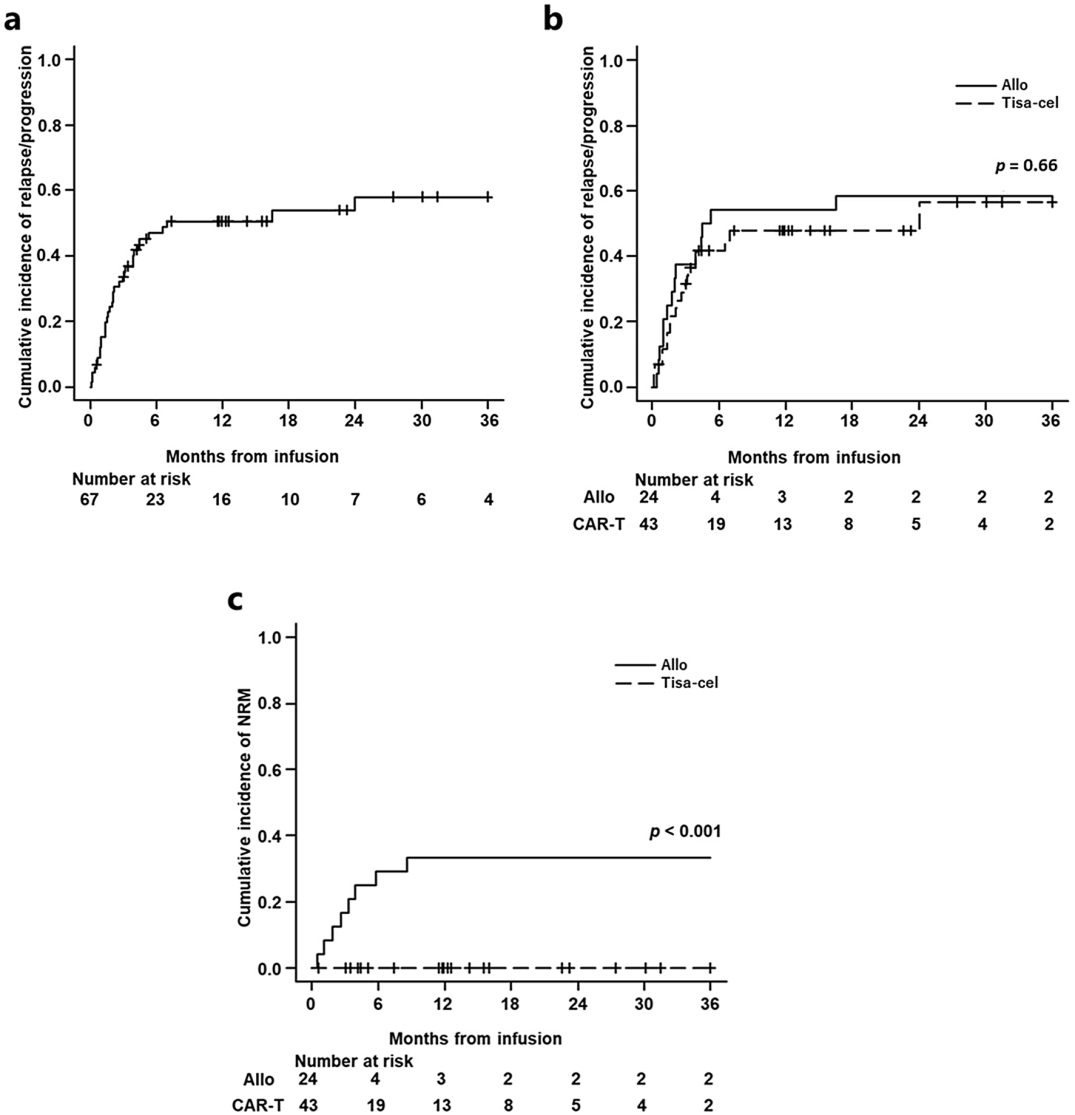
Relapse/progression rate in all LBCL (**a**). There was no significant difference in relapse/progression rate between the allo-SCT and tisa-cel groups (**b**). Although approximately one third of patients in the allo-SCT group died without relapse/progression, none of the patients in the tisa-cel group experienced NRM (**c**)

The one-year relapse/progression rate was 50.6% (95% CI: 37.4–62.4%) in the entire r/r LBCL cohort (Fig. 3a). The allo-SCT group showed similar one-year relapse/progression rates as the tisa-cel group (54.2% vs. 47.9%, 95% CI: 37.4–62.4% vs. 31.3–62.8%, *p* = 0.66) (Fig. 3b). There was no significant difference in the three-month relapse/progression rate between the allo-SCT and tisa-cel groups with PS ≥ 2 (allo-SCT vs. tisa-cel groups; 62.5% vs. 66.7%, 95% CI: 17.3–88.2% vs. 23.5–89.3%, *p* = 0.65), EN ≥ 2 (57.1% vs. 44.7%, 95% CI: 12.1–86.2% vs. 23.3–64.2%,* p* = 0.98), chemorefractory disease (50% vs. 45.8%, 95% CI: 23.3–71.9% vs. 25–64.4%, *p* = 0.74), and LDH ≥ ULN (42.1% vs. 36.8%, 95% CI: 19.5–63.3% vs. 18.1–55.7%, *p* = 0.76). In the univariate analysis of relapse/progression, chemorefractory disease was identified as an unfavorable factor (HR 3.5, 95% CI: 1.6–7.7, *p* = 0.0021). In multivariate analysis, tisa-cel showed no significant difference, whereas chemorefractory disease was significantly associated with an unfavorable prognosis (HR 3.2, 95% CI: 1.4–7.0, *p* = 0.0048) (Table 5).

**Table 5:** Univariate and multivariate analyses for relapse/progression

	Univariate			Multivariate		
	HR	95% CI	*P* value	HR	95% CI	*P* value
Treatment: Tisa-cel	0.79	0.40–1.5	0.48	0.87	0.69–3.2	0.3
Chemosensitivity: Chemorefractory disease	3.5	1.6–7.7	0.0021	3.2	1.4–7.0	0.0048
LDH ≥ ULN	2.0	0.97–4.1	0.061	1.5	0.43–1.7	0.70

*CI* confidence interval; *HR* hazard ratio; *LDH* lactate dehydrogenase; *OS* overall survival; *PFS* progression-free survival; *tisa-cel* tisagenlecleucel; *ULN* upper limit of normal

The one-year NRM in the entire r/r LBCL cohort was 12.9% (95% CI: 5.9–22.6%). Although the one-year NRM was 33.3% (95% CI: 15.1–52.9%) in the allo-SCT group, none of the patients in the tisa-cel group experienced NRM (*p* < 0.001) (Fig. 3c). Out of 24 allo-SCT patients, 12 patients each received MAC and RIC regimen. The one-year NRM was higher in the MAC group than in the RIC group (50.0% vs. 16.7%, 95% CI:17.9–75.7% vs. 1.9–44.4%, *p* = 0.06). The one-year NRM was similar between the patients treated with allo-SCT in 2003–2010 and in 2011–2020 (one-year NRM; 30.0% vs. 35.7%, 95% CI:5.2–61.2% vs. 11.3–61.5%, *p* = 0.75). In the allo-SCT group, a total of nine transplant-related deaths occurred. The cause of NRM was as follows: thrombotic microangiopathy (n = 2), acute graft-versus-host disease (n = 1), and infection (n = 1) in 2003–2010, respectively, and veno-occlusive disease (n = 1), infection (n = 1), progressive multifocal leukoencephalopathy (n = 1), interstitial pneumonia (n = 1), and unknown cause (n = 1) in 2011–2020, respectively.

### Comparison of outcomes between allo-SCT received RIC regimen and tisa-cel

Based on the above result that NRM is better in the patients with the RIC regimen than those with the MAC regimen, we additionally compared survival outcomes in patients who received allo-HCT with RIC regimen and tisa-cel. The allo-SCT with RIC regimen group had a significantly worse prognosis than the tisa-cel group (one-year PFS: 16.7% vs. 52.1%, 95% CI: 2.7–41.3% vs. 35.3–66.4%, *p* = 0.036, one-year OS: 25.0% vs. 61.1%, 95% CI: 6.0–50.5% vs. 43.0–75.0%, *p* = 0.046) (Supplemental Fig. 1a, b). The one-year relapse/progression rate was higher in the RIC group than the tisa-cel group (66.7% vs. 47.9%, 95% CI: 30.6–87.0% vs. 31.3–62.8%, *p* = 0.25), although not statistically significant (Supplemental Fig. 1c). The one-year NRM was 16.7% (95% CI: 1.9–44.4%) vs. 0% (95% CI: 0–0%) in the RIC group and tisa-cel group, respectively (*p* = 0.017) (Supplemental Fig. 1d).

### Comparison of outcomes between allo-SCT and tisa-cel after propensity score matching

To obtain more robust results and adjust the differences in patient background between the allo-SCT group and the tisa-cel group, we performed PSM based on the 1:1 matching ratio. At first, we chose the factors “age” and the “number of prior regimens” because these two factors differed significantly between the two groups (allo-SCT n = 18, tisa-cel n = 18) (Supplemental Table 1). Second, we chose the factors “LDH” and “chemosensitivity” because they were significantly associated with prognosis (allo-SCT n = 19, tisa-cel n = 19). The standardized differences were less than 0.1 for age and the number of prior regimens in first cohort, and less than 0.2 for LDH and chemosensitivity in second cohort. The area under the curve in the logistic regression model for the propensity score were 0.73 in first cohort and 0.64 in second cohort (Supplemental Fig. 2).

Using the first PSM cohort, the allo-SCT group had significantly worse survival outcomes than the tisa-cel group (median PFS: 2.0 vs. 24.0 months, 95% CI: 1.0–3.9 months vs. 2.6 months-NR, *p* < 0.001, and median OS: 3.6 months vs. NR, 95% CI: 2.5–6.7 months vs. 7.2 months-NR, *p* < 0.001) (Supplemental Fig. 3a, b). The allo-SCT group still showed similar relapse/progression rates as the tisa-cel group (one-year relapse/progression: 55.6 vs. 45.5%, 95% CI:28.6–75.9 vs. 21.1–67.1%, *p* = 0.27) (Supplemental Fig. 3c). The one-year NRM was 38.9% (95% CI: 15.8–61.7%) in the allo-SCT group, and none of the patients in the tisa-cel group experienced NRM (*p* = 0.004) (Supplemental Fig. 3d). In univariate and multivariate analysis, tisa-cel was still associated with a favorable survival outcome (Supplemental Table 2a, b). In addition, in univariate analysis, tisa-cel showed no significant difference, whereas chemorefractory disease was significantly associated with relapse/progression rate (HR 2.7, 95% CI: 1.1–7.1, *p* = 0.036). In multivariate analysis, chemorefractory disease was significantly associated with an unfavorable prognosis (HR 2.7, 95% CI: 1.1–6.9, *p* = 0.036) (Supplemental Table 2c).

Subgroup analysis was performed for patients with poor prognostic factors for tisa-cel in the context of the following parameters: PS ≥ 2 (all-SCT n = 6, tisa-cel n = 2), EN ≥ 2 (allo-SCT n = 5, tisa-cel n = 10), chemorefractory disease (allo-SCT n = 12, tisa-cel n = 10), and LDH ≥ ULN (allo-SCT n = 14, tisa-cel n = 12). Among the patients with poor prognostic factors, patients with EN ≥ 2, chemorefractory disease, or LDH ≥ ULN showed worse survival outcomes when receiving allo-SCT than tisa-cel (Supplemental Fig. 4a–f). Patients with PS ≥ 2 showed no significant differences in terms of PFS and OS between the allo-SCT and tisa-cel groups (Supplemental Table 3). Patients with EN ≥ 2 showed significantly worse relapse/progression rate in the allo-SCT group than the tisa-cel group (three-month relapse/progression: 80.0% vs. 40.0%, 95% CI: 41.8–99.2% vs. 17.3–74.7%, *p* = 0.04). There was also no significant difference in relapse/progression rate between the allo-SCT and tisa-cel groups with PS ≥ 2, chemorefractory disease, and LDH ≥ ULN. (Supplemental Fig. 5a, c, e). No one experienced NRM with EN ≥ 2. Approximately one-third of patients with chemorefractory or LDH ≥ ULN in the allo-SCT group died without relapse/progression (Supplemental Fig. 5b, d, f).

Using the second PSM cohort, the allo-SCT group had also significantly worse survival outcomes than the tisa-cel group (median PFS: 2.6 vs. 24.0 months, 95% CI: 1.1–5.3 months vs. 2.1 months-NR, *p* = 0.017, and median OS: 4.6 months vs. NR, 95% CI: 1.8–8.1 months vs. 7.2 months-NR, *p* = 0.014). (Supplemental Fig. 6a, b). The allo-SCT group showed similar relapse/progression rates as the tisa-cel group (one-year relapse/progression: 47.4 vs. 45.9%, 95% CI:23.2–68.2 vs. 23.7–68.6%, *p* = 0.98) (Supplemental Fig. 6c). The one-year NRM was 36.8% (95% CI: 15.3–58.8%) in the allo-SCT group, and none of the patients in the tisa-cel group experienced NRM (*p* = 0.005) (Supplemental Fig. 6d).

Collectively, we analyzed the outcomes of the allo-SCT and tisa-cel again using PSM. The results were generally similar to those before both PSM, and tisa-cel was associated with a favorable prognosis.

## Discussion

In the present study, we compared the outcomes of patients with r/r LBCL who received either allo-SCT or tisa-cel in view of the poor prognostic factors associated with CAR-T-cell therapy. Overall, patients with poor prognostic factors showed dismal outcomes, irrespective of the type of cellular therapy. However, although survival outcomes for patients with PS ≥ 2 or EN ≥ 2 were similar between the two cellular therapy cohorts, those with chemorefractory disease or LDH ≥ ULN who were treated with tisa-cel had a better prognosis than those treated with allo-SCT. This result was re-confirmed among the PSM cohort. Our results suggest that even in patients with poor prognostic factors for CAR-T cell therapy, there is no rationale for early allo-SCT before tisa-cel.

Previous studies comparing allo-SCT and CAR-T cell therapies reported no significant differences in PFS and OS [10, 18, 19]. Contrarily, we found better survival outcomes associated with CAR T-cell therapy in this study. Previous reports showed that the one-year OS in patients treated with tisa-cel ranged between 46.3 and 49%, which was inferior to the results of our CAR-T group (one-year OS 61.1%) [4, 12, 26]. However, the three-year OS of patients treated with allo-SCT at our institution (16.7%) was lower than that reported in previous studies (19–45%) [10, 27–30]. It is known that patients who are chemorefractory disease, have LDH ≥ ULN, and have received more than four prior regimens have inferior survival outcomes associated with allo-SCT [10, 27, 31, 32]. In this study, majority of the patients treated with allo-SCT were chemorefractory disease, had LDH ≥ ULN, and received more prior treatment regimens. In addition, previous reports have shown that RIC regimen is recommended for allo-SCT in DLBCL because NRM increases in MAC group, particularly older LBCL patients [33]. However, in this analysis, half of the patients in allo-SCT group received MAC regimen and NRM was higher in the MAC group than in the RIC group. These differences in patient, historical backgrounds, and high intensity conditioning regimen may have contributed to the poor prognosis associated with allo-SCT. When comparing patients who received RIC regimen or tisa-cel, although NRM was decreased in the allo-SCT group, relapse/progression rate was increased and the patients in RIC group had poor prognosis than tisa-cel group.

In the context of the four poor prognostic factors, patients who received tisa-cel with chemorefractory disease or LDH ≥ ULN showed better PFS and OS than those who received allo-SCT. However, the relapse rate in patients with these two factors was similar between the tisa-cel and allo-SCT groups, and the advantages of tisa-cel over allo-SCT disappeared (Table 2c). These findings suggest that a high tumor burden before cellular therapy, rather than the type of cellular therapy, strongly affects the relapse rate. In the context of toxicity, conditioning therapy before allo-SCT, which is more intensive than lymphodepletion treatment before CAR-T cell therapy, tends to increase toxicity. This toxicity partially contributed to the high NRM in the allo-SCT group and a worse prognosis in terms of PFS and OS. The results were generally similar to those after PSM with chemorefractory disease or LDH ≥ ULN, and tisa-cel was associated with a favorable prognosis.

In the context of PS ≥ 2 and EN ≥ 2, the relapse rate, PFS, and OS were similar between the tisa-cel and allo-SCT groups. Although the NRM in the allo-SCT group was higher compared to the tisa-cel group, the number of patients in the group with PS ≥ 2 or EN ≥ 2 was relatively small (n = 8 and 7, respectively). However, among the first PSM cohort, the relapse/progression was significantly higher in the allo-SCT group with EN ≥ 2 and all patients in the allo-SCT group relapsed within 5 months. This is an encouraging preliminary result to use of tisa-cel in patients with multiple EN lesions. However, a larger cohort size could validate our results and improve prognostic value in this subset of patients.

Survival outcomes after relapse post-cellular therapy in patients with poor prognostic factors were found to be poor in this study, with 1.6 and 4.6 months in the allo-SCT groups and tisa-cel groups, respectively. This finding indicates that patients at risk of relapse show dismal outcomes after cellular therapy failure. A study reported that an effective therapy for post-CAR-T cell relapse with the median OS from the first post-CAR-T treatment being 8 months [34]. However, novel agents such as polatuzumab vedotin or lenalidomide have been reported to improve the outcomes after CAR-T cell failure [35]. Comprehensively, our results showed that the patients with a risk of relapse required better consolidation therapy or salvage therapy to prevent the recurrence or to improve post-recurrence therapy outcomes irrespective of the cellular therapy.

This study had a few limitations that need consideration. First, this is a retrospective analysis, and the study only included patients from a single center, which may have limited its statistical power and introduced important differences in outcomes. In addition, there were many differences in patient characteristics between the allo-SCT group and the tisa-cel group. However, we additionally performed PSM based on age, the number of prior regimens, LDH, and chemosensitivity. We attempted to include four factors (age, the number of prior regimens, LDH level, and chemosensitivity at the time of infusion/transplantation) in the PSM at the same time, but could not properly adjust for patient background. Therefore, this analysis was not performed. Second, the outcomes of allo-SCT have been reported to improve with the development of therapeutic drugs and transplantation methods [15, 36, 37]. The recent previous reports described that the one-year NRM after allo-SCT for DLBCL was 13–47% [10, 16, 19]. While we understand that our study included a small number of patients, and most patients received allo-SCT around the year 2010, the NRM rate in our study was similar to the above studies that compared allo-SCT to CAR-T therapy. Third, the CAR-T therapy expanding, the number of allo-SCT was decreased [17]. Therefore, the date of allo-SCT and CAR-T therapy was not contemporaneous with our study. Fourth, to avoid heterogeneity, we excluded liso-cel and axi-cel. However, axi-cel is considered more effective than tisa-cel [26]. Fifth, in this analysis, the number of patients is smaller than in previous reports [10, 18, 19]. However, they did not compare whether CAR-T cell therapy is more effective or less toxic than allo-SCT in patients with poor prognostic factors. Therefore, our data will be a reference for the treatment strategy for such patients.

In conclusion, even in patients with poor prognostic factors for CAR-T cell therapy, tisa-cel showed better outcomes than allo-SCT. However, these patients had dismal outcomes, irrespective of the type of cellular therapy. Therefore, the development of safe and effective before and after cellular therapy is warranted.

## Supplementary Information

Below is the link to the electronic supplementary material.

Supplementary file1 (DOCX 39 KB)

Supplementary file2 (JPG 312 KB)

Supplementary file3 (JPG 499 KB)

Supplementary file4 (JPG 301 KB)

Supplementary file5 (JPG 422 KB)

Supplementary file6 (JPG 410 KB)

Supplementary file7 (JPG 349 KB)

## Data Availability

The datasets generated during and/or analyzed during the current study are available from Kenta Hayashino or Toshiki Terao on reasonable request.
