# Supplementary material for: Outcomes of allogeneic SCT versus tisagenlecleucel in patients with R/R LBCL and poor prognostic factors
Source: Int J Hematol. 2024 Dec 16;121(2):232–43. doi: 10.1007/s12185-024-03888-9 (PMC11782353; doi:10.1007/s12185-024-03888-9)
Supplement: Supplementary file 1 — Supplementary file1 (DOCX 39 KB) [file 12185_2024_3888_MOESM1_ESM.docx]

**Supplemental Data**

**Title**

**Comparison of Outcomes of Allogeneic HSCT vs. Tisagenlecleucel in Patients with R/R LBCL and Poor Prognostic Factors**

Kenta Hayashino^1^, Toshiki Terao^1, 2^**^＊^**, Hisakazu Nishimori^1,3^, Wataru Kitamura^1,2^, Hiroki Kobayashi^1,2^, Chihiro Kamoi^4^, Keisuke Seike^1^, Hideaki Fujiwara^1^, Noboru Asada^1^, Daisuke Ennishi^5^, Keiko Fujii^1,6^, Nobuharu Fujii^1,4^, Ken-ichi Matsuoka^1^, Yoshinobu Maeda^1^

^1^ Department of Hematology and Oncology, Okayama University Hospital, 2-5-1 Shikata, Okayama-shi, Okayama, Japan

^2^ Department of Hematology, Oncology and Respiratory Medicine, Okayama University Graduate School of Medicine, Dentistry and Pharmaceutical Sciences, Okayama, Japan

^3^ Department of Hematology, Hiroshima City Hiroshima Citizens Hospital, Hiroshima, Japan

^4^ Division of Blood Transfusion, Okayama University Hospital, Okayama, 2-5-1 Shikata, Okayama-shi, Japan

^5^ Center for Comprehensive Genomic Medicine, Okayama University Hospital, 2-5-1 Shikata, Okayama-shi, Okayama, Japan

^6^ Division of Clinical Laboratory, Okayama University Hospital, Okayama, 2-5-1 Shikata, Okayama-shi, Japan

**Supplemental Table.1 Survival outcomes with poor prognostic factors after PSM**

|  | Allo-SCT (n = 18) | Tisa-cel (n = 18) | *P* value | Standard difference |
| --- | --- | --- | --- | --- |
| Median age, y (range) | 57 (47-72) | 56.5 (45-69) | 0.72 | 0.198 |
| 40-49 | 2 (11.1) | 3 (16.7) | 1 | < 0.01 |
| 50-59 | 9 (50) | 8 (44.4) |  |  |
| 60-69 | 6 (33.3) | 7 (38.9) |  |  |
| 70- | 1 (5.6) | 0 |  |  |
| Gender, n (%) |  |  | 1 | < 0.01 |
| Male | 10 (55.6) | 10 (55.6) |  |  |
| Female | 8 (44.4) | 8 (44.4) |  |  |
| Disease histology, n (%) |  |  | 0.79 | 0.365 |
| DLBCL-NOS | 9 (50) | 12 (66.7) |  |  |
| Transformed | 6 (33.3) | 4 (22.2) |  |  |
| Immunodeficiency associated | 1 (5.6) | 1 (5.6) |  |  |
| Other | 2 (11.1) | 1 (5.6) |  |  |
| Stage, at diagnosis, n (%) |  |  | 0.1 | 0.76 |
| StageⅠ/Ⅱ | 0 | 4 (22.2) |  |  |
| StageⅢ/Ⅳ | 18 (100) | 14 (77.8) |  |  |
| IPI at diagnosis, n (%) |  |  | 0.29 | 0.64 |
| Low/Low-Int | 9 (50) | 7 (38.9) |  |  |
| High-Int/High | 9 (50) | 8 (44.4) |  |  |
| N/A | 0 | 3 (16.7) |  |  |
| PS at infusion |  |  | 0.23 | 0.56 |
| 0,1 | 12 (66.7) | 16 (88.9) |  |  |
| 2 ≥ | 6 (33.3) | 2 (11.1) |  |  |
| Median No. of prior regimen |  |  | 1 | < 0.01 |
| 1-3 | 3 (16.7) | 3 (16.7) |  |  |
| 4- | 15 (83.3) | 15 (83.3) |  |  |
| Previous history of autologoous-SCT, n (%) | 6 (33.3) | 9 (50) | 0.5 | 0.34 |
| CNS invasion, n (%) | 3 (16.7) | 3 (16.7) | 1 | < 0.01 |
| Bone marrow invasion, n (%) | 11 (61.1) | 6 (33.3) | 0.18 | 0.58 |
| EN lesion ≥ 2, n (%) | 5 (27.8) | 10 (55.6) | 0.18 | 0.59 |
| LDH ≥ ULN, n (%) | 14 (77.8) | 12 (66.7) | 0.71 | 0.25 |
| Disease status at infusion, n (%) |  |  | 0.69 | 0.36 |
| CR | 3 (16.7) | 5 (27.8) |  |  |
| Relapse | 10 (55.6) | 7 (38.9) |  |  |
| Primary refractory | 5 (27.8) | 6 (33.3) |  |  |
| Response for last chemotherapy, n (%) |  |  | 0.73 | 0.23 |
| Chemosensitive | 6 (33.3) | 8 (44.4) |  |  |
| Chemorefractory | 12 (66.7) | 10 (55.6) |  |  |
| Conditioning regimen, n (%) |  |  | N/A | N/A |
| MAC | 8 (44.4) | N/A |  |  |
| RIC | 10 (55.6) | N/A |  |  |
| Stem cell source, n (%) |  |  | N/A | N/A |
| BM | 4 (22.2) | N/A |  |  |
| PB | 8 (44.4) | N/A |  |  |
| CB | 6 (33.3) | N/A |  |  |
| Donor relation |  |  | N/A | N/A |
| Matched related | 2 (11.1) | N/A |  |  |
| Mismatched related | 6 (33.3) | N/A |  |  |
| Matched unrelated | 3 (16.7) | N/A |  |  |
| Mismatched unrelated | 7 (38.8) | N/A |  |  |
| Year of allo-HSCT or tisa-cell, n (%) |  |  | < 0.001 | 1.7 |
| 2003-2010 | 6 (33.3) | 0 |  |  |
| 2011-2020 | 12 (66.7) | 9 (50) |  |  |
| 2021-2023 | 0 | 9 (50) |  |  |
| Median follow up time in survivors, months (range) | 80.2 (80.0-80.3) | 29.2 (3.5-40.6) | 0.03 | 6.6 |

Allo-SCT, allogeneic hematopoietic stem cell transplantation; auto-SCT, autologous hematopoietic stem cell transplantation; CNS, central nerve system; CR, complete response; DLBCL-NOS, diffuse large B-cell lymphoma not otherwise specified; EN, extranodal; IPI, International Prognostic Index; LDH, lactate dehydrogenase; N/A, not available; MAC, myeloablative conditioning; PS, performance status; RIC, reduced intensity conditioning; tisa-cel, tisagenlecleucel; ULN, upper limit of normal

**Supplemental Table.2a Univariate and multivariate analyses for PFS after PSM**

|  | Univariate | | | Multivariate | | |
| --- | --- | --- | --- | --- | --- | --- |
|  | HR | 95% CI | *P* value | HR | 95% CI | *P* value |
| Treatment: Tisa-cel | 0.23 | 0.1-0.54 | 0.00074 | 0.23 | 0.094-0.55 | 0.001 |
| Chemosensitivity: Chemorefractory disease | 2.4 | 1-5.5 | 0.043 | 2.3 | 0.94-5.4 | 0.069 |
| LDH ≥ ULN | 1.3 | 0.55-2.9 | 0.59 | 1.14 | 0.49-2.7 | 0.76 |

**Supplemental Table.2b Univariate and multivariate analyses for OS after PSM**

|  | Univariate | | | Multivariate | | |
| --- | --- | --- | --- | --- | --- | --- |
|  | HR | 95% CI | *P* value | HR | 95% CI | *P* value |
| Treatment: Tisa-cel | 0.24 | 0.1-0.57 | 0.0013 | 0.21 | 0.084-0.53 | 0.001 |
| Chemosensitivity: Chemorefractory disease | 3.76 | 1.4-10.2 | 0.0096 | 4.5 | 1.5-13.5 | 0.007 |
| LDH ≥ ULN | 1.46 | 0.58-3.7 | 0.42 | 0.84 | 0.32-2.2 | 0.73 |

**Supplemental Table 2c Univariate and multivariate analyses for relapse/progression after PSM**

|  | Univariate | | | Multivariate | | |
| --- | --- | --- | --- | --- | --- | --- |
|  | HR | 95% CI | *P* value | HR | 95% CI | *P* value |
| Treatment: Tisa-cel | 0.66 | 0.28-1.5 | 0.34 | 0.71 | 0.3-1.7 | 0.44 |
| Chemosensitivity: Chemorefractory disease | 2.7 | 1.1-7.1 | 0.036 | 2.7 | 1.1-6.9 | 0.036 |
| LDH ≥ ULN | 1.1 | 0.45-2.6 | 0.87 | 0.93 | 0.34-2.6 | 0.88 |

CI, confidence interval; HR, hazard ratio; LDH, lactate dehydrogenase; OS, overall survival; PFS, progression-free survival; tisa-cel, tisagenlecleucel; ULN, upper limit of normal

**Supplemental Table.3 Survival outcomes with poor prognostic factors after PSM**

|  | Number of patients | Median PFS (month, 95% CI) | *P* value | Median OS (month, 95% CI) | *P* value | Three-month relapse/ progression (%, 95% CI) | *P* value | One-year NRM (%, 95% CI) | *P* value |
| --- | --- | --- | --- | --- | --- | --- | --- | --- | --- |
| All cases |  |  |  |  |  |  |  |  |  |
| Allo-SCT | 18 | 2.0 (1.0-3.9) |  | 3.6 (2.5-6.7) |  | 44.4 (20.6-65.9) |  | 38.9 (15.8-61.7) |  |
| Tisa-cel | 18 | 24.0 (2.6-NR) | < 0.001 | NR (7.2-NR) | < 0.001 | 27.8 (9.7-50.0) | 0.27 | 0.0 (0.0-0.0) | 0.004 |
| PS ≥ 2 |  |  |  |  |  |  |  |  |  |
| Allo-SCT | 6 | 1.9 (0.66-NR) |  | 3.6 (0.95-NR) |  | 83.3 (8.6-98.7) |  | N/A (N/A-N/A) |  |
| Tisa-cel | 2 | 1.8 (1.6-NR) | 0.99 | 3.7 (2.2-NR) | 0.62 | N/A (N/A-N/A) | 0.99 | 0.0 (0.0-0.0) | 0.63 |
| EN ≥ 2 |  |  |  |  |  |  |  |  |  |
| Allo-SCT | 5 | 2.0 (0.95-NR) |  | 2.7 (0.95-NR) |  | 80.0 (41.8-99.2) |  | No NRM occurred in both groups | |
| Tisa-cel | 10 | 3.5 (1.3-24.0) | 0.054 | 8.2 (2.2-NR) | 0.016 | 40.0 (17.3-74.7) | 0.042 |  |  |
| Chemorefractory disease |  |  |  |  |  |  |  |  |  |
| Allo-SCT | 12 | 2.0 (0.95-3.9) |  | 3.6 (0.95-6.7) |  | 58.3 (24.7-81.2) |  | N/A (N/A-N/A) |  |
| Tisa-cel | 10 | 3.5 (1.3-NR) | 0.035 | 8.2 (2.2--NR) | 0.0047 | 40.0 (10.8-68.5) | 0.50 | 0.0 (0.0-0.0) | 0.058 |
| LDH ≥ ULN |  |  |  |  |  |  |  |  |  |
| Allo-SCT | 14 | 1.9 (0.95-3.9) |  | 3.3 (0.95-8.1) |  | 50.0 (21.2-73.4) |  | 35.7 (10.5-62.4) |  |
| Tisa-cel | 12 | NR (2.1-NR) | 0.0013 | NR (6.2-NR) | 0.0023 | 25.0 (5.4-51.7) | 0.21 | 0.0 (0.0-0.0) | 0.029 |

Allo-SCT, allogeneic hematopoietic stem cell transplantation; EN, extranodal; LDH, lactate dehydrogenase; N/A, not available; NR, not reached; NRM, non-relapse mortality; OS, overall survival; PFS, progression free survival; PS, performance status; tisa-cel, tisagenlecleucel; ULN, upper limit of normal

**Supplemental Figure Legend**

**Supplemental Fig. 1**

Comparison of outcomes between allo-SCT received RIC regimen and tisa-cel. Patients treated with tisa-cel had significantly better PFS (a) and OS (b). The relapse/progression rate was higher in the RIC group than the tisa-cel group (not statistically significant) (c). The NRM in the RIC group was still significantly higher than the tisa-cel group (d).

**Supplemental Fig. 2**

Patient flow diagram for PSM analysis.

**Supplemental Fig. 3**

After first propensity score matching, patients treated with tisa-cel had significantly better PFS (a) and OS (b). There was no significant difference in relapse/progression rate between the allo-SCT and tisa-cel groups (c). Although approximately one-third of patients in the allo-SCT group died without relapse/progression, none of the patients in the tisa-cel group experienced NRM (d).

**Supplemental Fig. 4**

Survival outcomes with poor prognostic factors for tisa-cel cell therapy after propensity score matching. The patient treated with tisa-cel had better PFS (a) and OS (b) with EN ≥ 2, PFS (c) and OS (d) with chemorefractory, and PFS (e) and OS (f) with LDH ≥ ULN.

**Supplemental Fig. 5**

Patients with EN ≥ 2 showed significantly worse relapse/progression rate in the allo-SCT group than the tisa-cel group (a). There was no significant difference in relapse/progression rate between the allo-SCT and tisa-cel groups with chemorefractory (c) and LDH ≥ ULN (e). No one experienced NRM with EN ≥ 2 (b). Approximately one-third of patients with chemorefractory (d) and LDH ≥ ULN (f) in the allo-SCT group experienced NRM.

**Supplemental Fig. 6**

After second propensity score matching, patients treated with tisa-cel had also significantly better PFS (a) and OS (b). There was no significant difference in relapse/progression rate between the allo-SCT and tisa-cel groups (c). Approximately one-third of patients in the allo-SCT group died without relapse/progression (d).
