# Supplementary figures and images for: Outcomes of allogeneic SCT versus tisagenlecleucel in patients with R/R LBCL and poor prognostic factors
Source: Int J Hematol. 2024 Dec 16;121(2):232–43. doi: 10.1007/s12185-024-03888-9 (PMC11782353; doi:10.1007/s12185-024-03888-9)

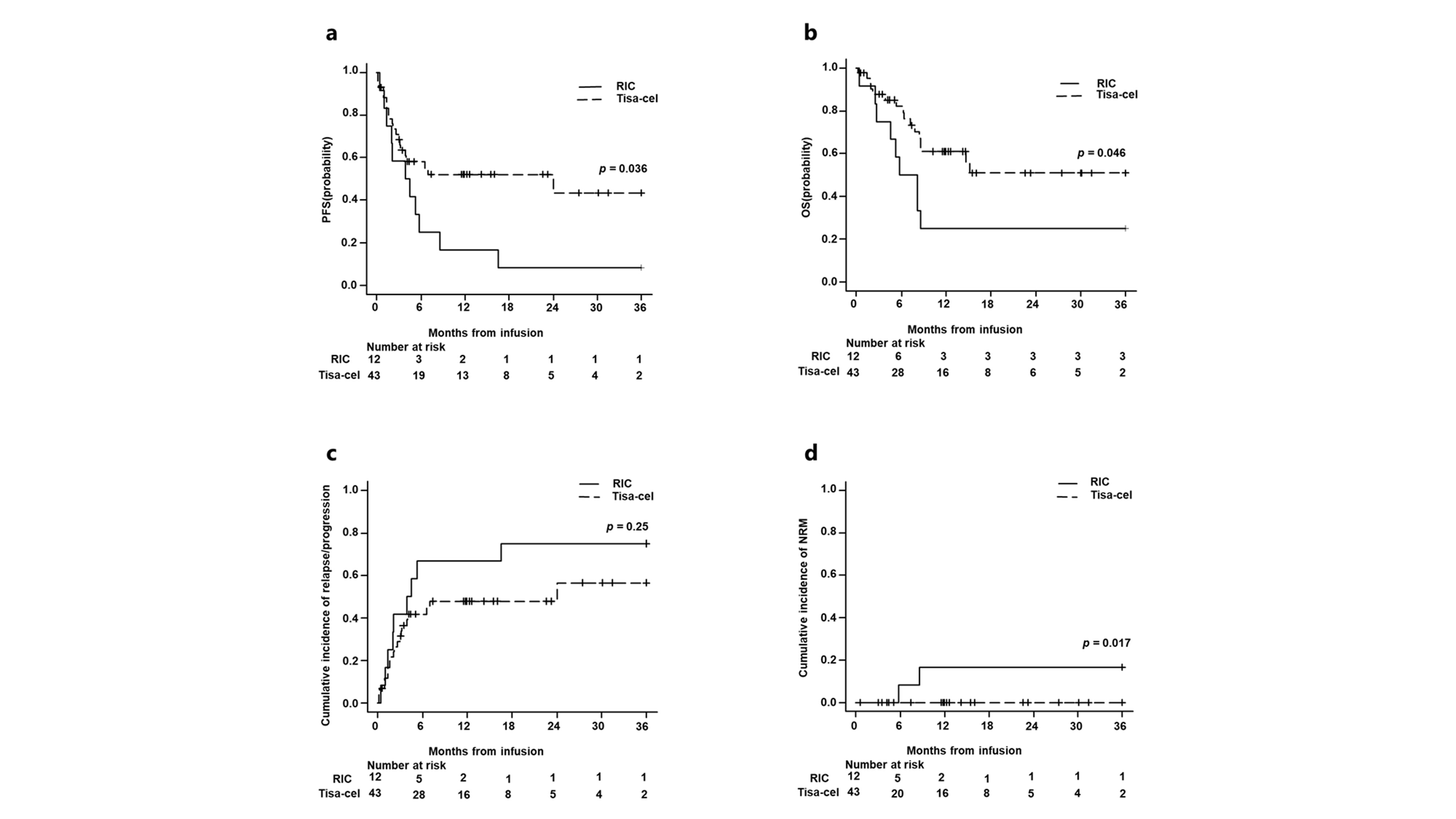

Supplement: Supplementary file 2 — Supplementary file2 (JPG 312 KB) [file 12185_2024_3888_MOESM2_ESM.jpg]

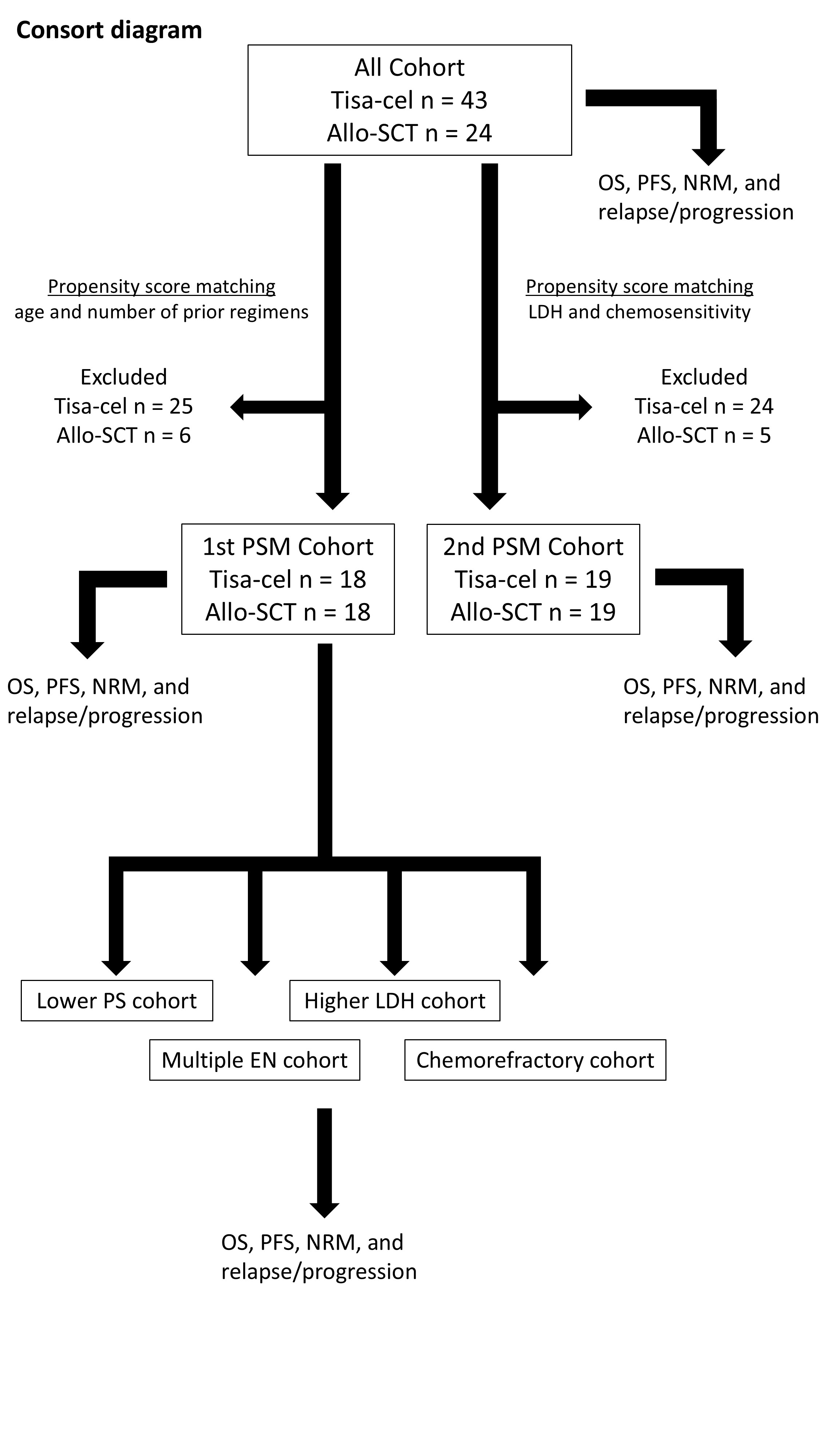

Supplement: Supplementary file 3 — Supplementary file3 (JPG 499 KB) [file 12185_2024_3888_MOESM3_ESM.jpg]

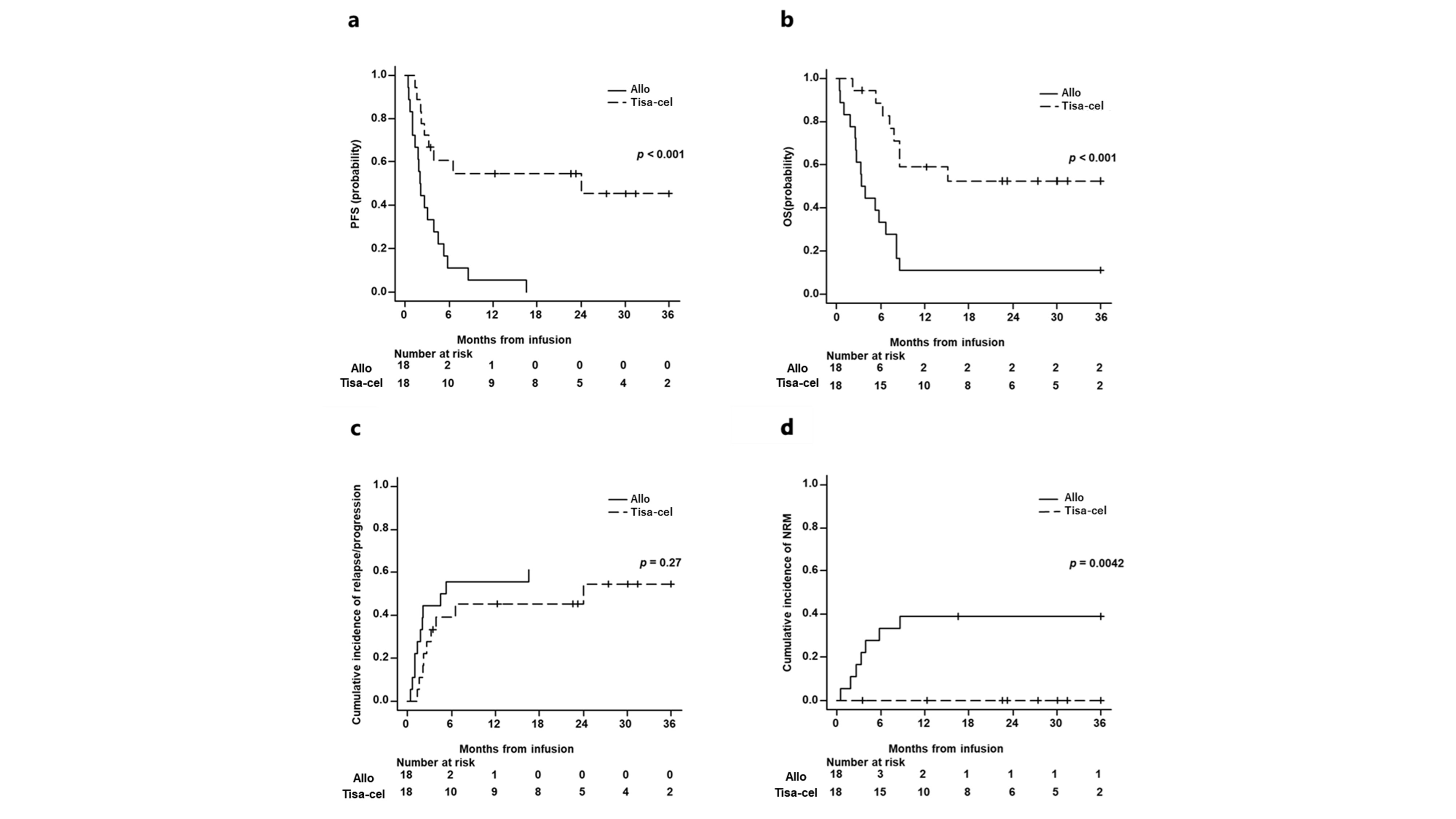

Supplement: Supplementary file 4 — Supplementary file4 (JPG 301 KB) [file 12185_2024_3888_MOESM4_ESM.jpg]

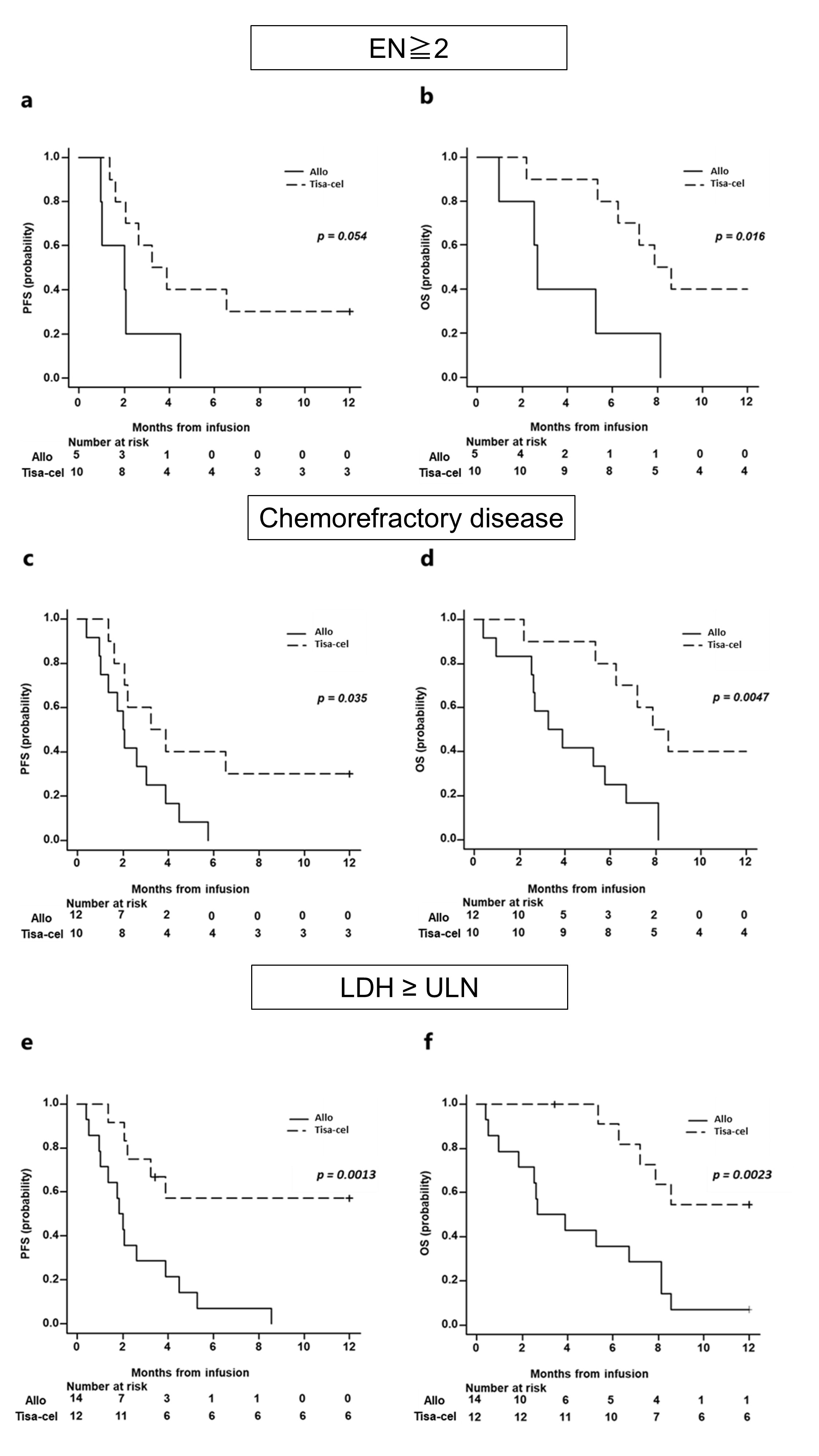

Supplement: Supplementary file 5 — Supplementary file5 (JPG 422 KB) [file 12185_2024_3888_MOESM5_ESM.jpg]

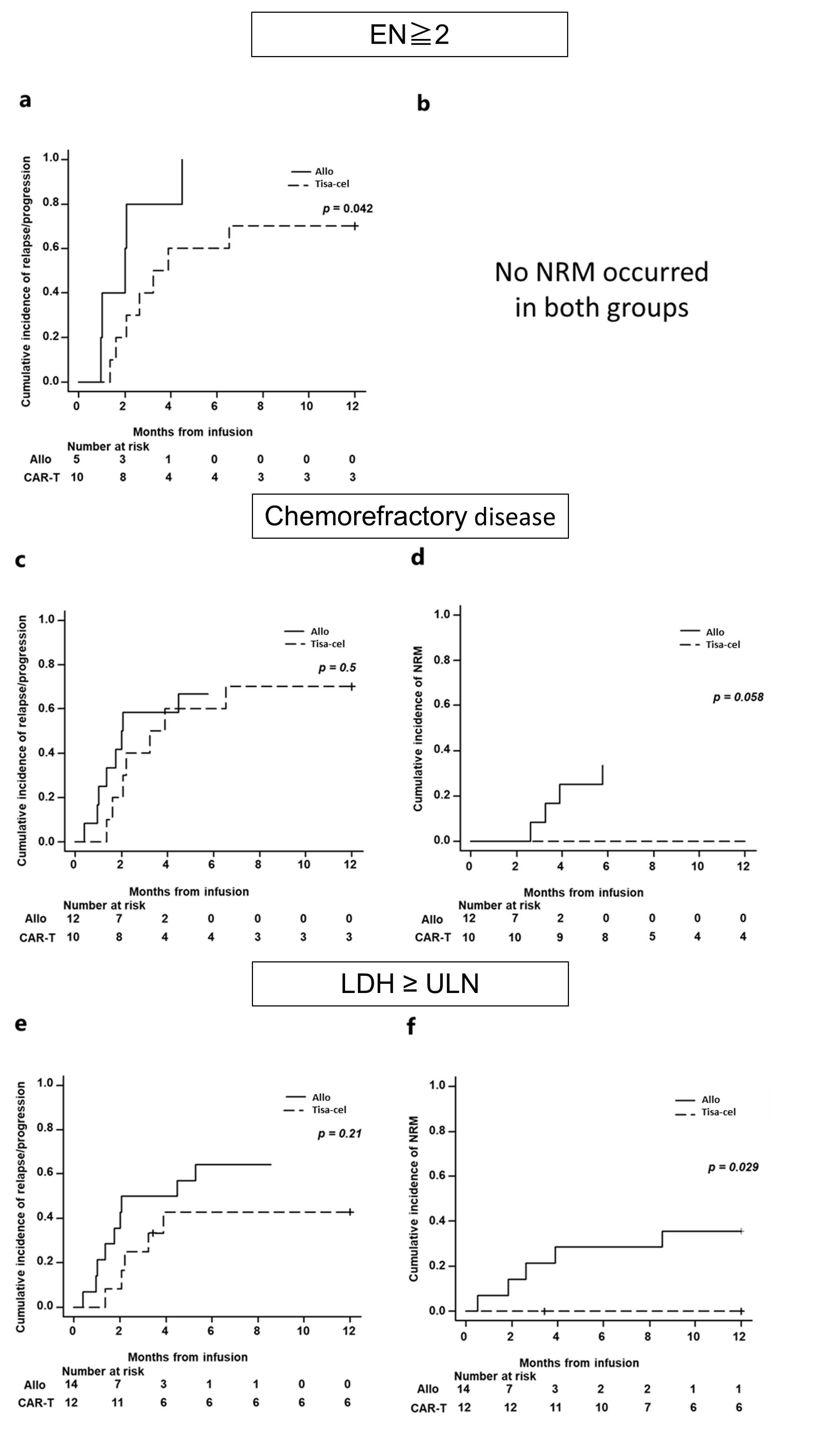

Supplement: Supplementary file 6 — Supplementary file6 (JPG 410 KB) [file 12185_2024_3888_MOESM6_ESM.jpg]

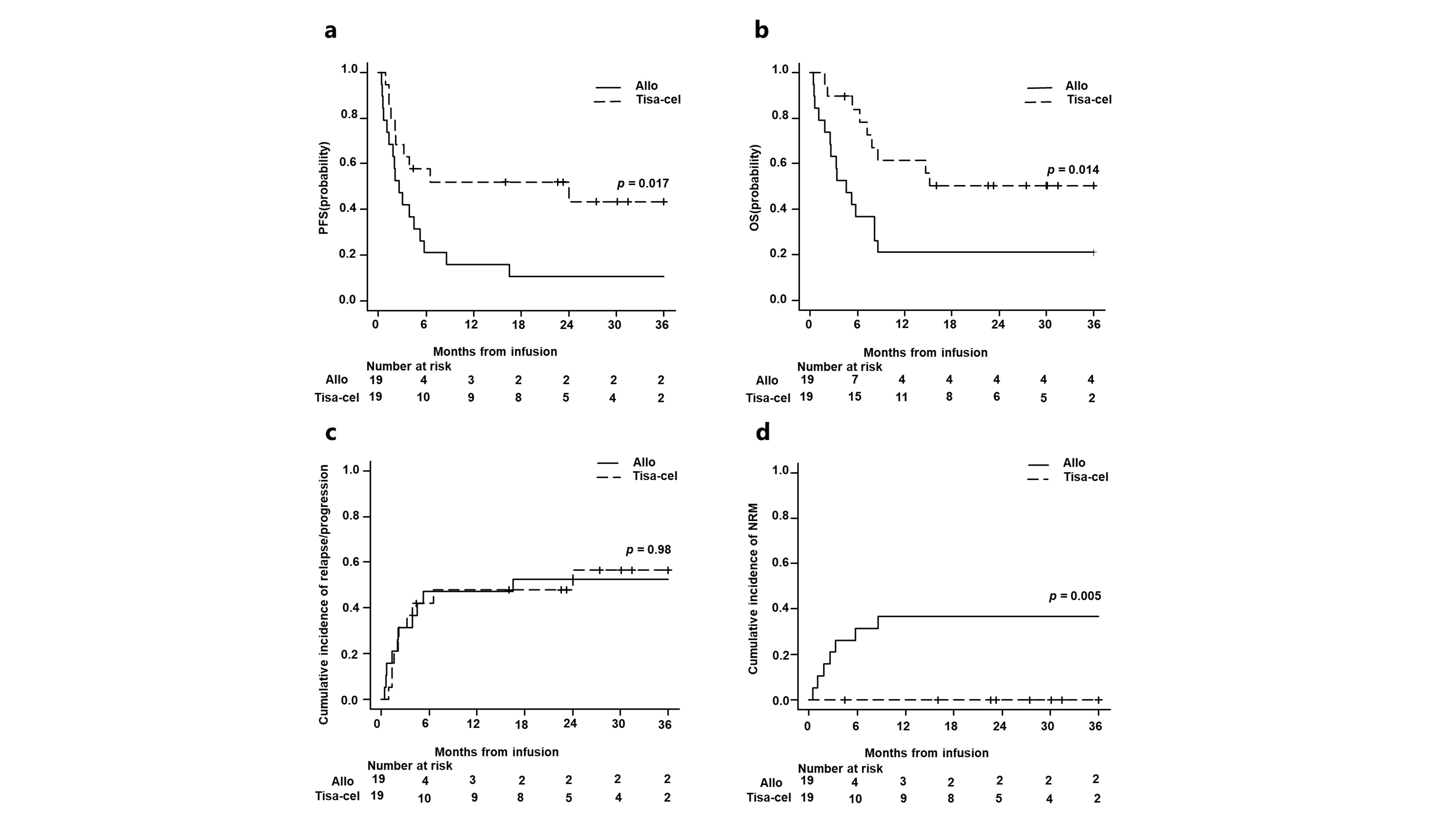

Supplement: Supplementary file 7 — Supplementary file7 (JPG 349 KB) [file 12185_2024_3888_MOESM7_ESM.jpg]
